# Using the National Early Warning Score (NEWS/NEWS 2) in different Intensive Care Units (ICUs) to predict the discharge location of patients

**DOI:** 10.1186/s12889-019-7541-3

**Published:** 2019-09-05

**Authors:** Hassan Zaidi, Mohamed Bader-El-Den, James McNicholas

**Affiliations:** 1 Centre for Healthcare Modelling and Informatics, University of Portsmouth, Portsmouth, UK; 2 School of Computing, University of Portsmouth, Portsmouth, UK; 3 Portsmouth Hospitals NHS Trust, Portsmouth, UK

**Keywords:** Patient discharge, Discharge location, Patient outcomes, Intensive care unit, National early warning score, Vital signs, Decision making, Outcome prediction

## Abstract

**Background:**

The National Early Warning Score (NEWS/NEWS 2) has been adopted across the National Health Service (NHS) in the U.K. as a method of escalating care for deteriorating patients. Intensive Care Unit (ICU) resources are limited and in high demand, with patient discharge a focal point for managing resources effectively. There are currently no universally accepted methods for assessing discharge of patients from an ICU, which can cause premature discharges and put patients at risk of subsequent deterioration, readmission to ICU or death.

**Methods:**

We tested the ability of the NEWS to discriminate patients within 24h of admission to an ICU in a U.S. hospital during 2001–2012, by their end discharge location: home; hospital ward; nursing facility; hospice and death. The NEWS performance was compared across five different ICU specialties, using the area under the receiver operating characteristic (AUROC) curve and a large vital signs database (*n*=2,723,055) collected from 28,523 critical care admissions.

**Results:**

The NEWS AUROC (95% CI) at 24h following admission: all patients 0.727 (0.709–0.745); Coronary Care Unit (CCU) 0.829 (0.821–0.837); Cardiac Surgery Recovery Unit (CSRU) 0.844 (0.838–0.850); Medical Intensive Care Unit (MICU) 0.778 (0.767–0.791); Surgical Intensive Care Unit (SICU) 0.775 (0.762–0.788); Trauma Surgical Intensive Care Unit (TSICU) 0.765 (0.751–0.773).

**Conclusions:**

The NEWS has reasonable discrimination for any ICU patient’s discharge location. The NEWS has greater ability to discriminate patients in the Coronary Care Unit (CCU) and Cardiac Surgery Recovery Unit (CSRU) compared to other ICU specialties. The NEWS has the real potential to be applied within a universal discharge planning tool for ICU, improving patient safety at the point of discharge.

## Background

### The NEWS

The National Early Warning Score (NEWS / NEWS 2) has now been widely implemented by the National Health Service (NHS) in the United Kingdom (U.K). The NEWS was created to standardise the process of responding to clinical deterioration, using six physiological parameters (with an additional weighting for supplemental oxygen) in acutely ill patients. The NEWS is used to track the clinical condition of patients and to trigger a clinical response [1]. Each of the physiological NEWS parameters is allocated a score according to the magnitude of disturbance to each parameter. The individual parameter scores are totalled to form the aggregate NEWS for the patient, which is then used for determining clinical responses and any escalation of care.

### ICU demand & the discharge problem

Intensive care resources are limited and expensive commodities, therefore managing bed flow is vital [2] to ensure high quality of care to those patients who need it. Appropriate and timely discharge is one of the means by which pressure for beds in the Intensive Care Unit (ICU) may be mitigated [3]. However, for 856 discharges in an 11-bed ICU over a 16 month period, it was found that up to 1 in 6 discharges from the ICU were unsuccessful in the first attempt, which could have been due to medical deterioration, level of care issues or administrative problems [4].

In a separate 22-bed ICU, covering 652 discharges over a 6-month period, up to 81% [5] of delayed ICU discharges were due to a lack of available beds in hospital wards for transfer of ICU patients. This type of discharge delay often results in other, sicker patients being unable to be admitted until a recovering ICU patient leaves [6, 7].

With limited ICU beds and increasing demand for admissions, patients are sometimes discharged by triage [2, 8] instead of through a review process by attending physicians with collaboration from other ICU care team members [9]. Incorrect triage discharges pose additional risks for the patient, [10] and some ICU patients are discharged out of hours, [11, 12] despite findings that discharges at night have been associated with increased mortality [10, 12].

### Current ICU discharge methods

Discharge planning is usually by consensus and suffers from a lot of variability in the clinical decision-making processes [9, 13]. The majority of ICUs do not use written patient discharge guidelines [2]. Clinicians have rather little secure evidence upon which to base any decision about discharge location [14, 15].

This ambiguity has the potential to lead to poor management of patients, which can result in premature discharge and subsequently death or readmission [2, 16]. This has been a factor in the motivation to create critical care outreach teams and triage models [2, 16–18] in order to improve discharge outcomes.

### The NEWS for discharge planning

Evidence-based discharge guidelines are necessary to safeguard the ICU patient discharge practice. We illustrate how the NEWS could be used in an empirical discharge plan to discriminate by discharge location within the first 24h of admission to ICU; within ICU specialties as well as for all critical care patients. The likely benefits associated with an empirical predictive discharge plan include: allowing providers to have earlier knowledge of upcoming patient demands; improve resource allocation; reduce bed pressures; reducing the risk and incidence of premature discharge; increasing the overall efficiency of the discharge process and reducing discharge delays.

We selected the first 24h of admission to ICU, as early prediction permits planning. If we were to use readings closer to a patients discharge, such as from day-2 onwards, then clinically it would be already quite apparent where the patient would be discharged to and confers no planning advantage. For example, if a patient has fully recovered consciousness following their out-of-hospital cardiac arrest on day-4 of their stay, then they would be discharged to the ward and then on to home. If that were known on day-1, then the ward and the ICU could plan activity more confidently.

## Methods

### Data

This was a retrospective study utilising surgical (SICU), coronary (CCU), cardiac surgery recovery (CSRU), medical (MICU) and trauma surgical (TSICU) intensive care patients with a single complete admission (i.e. patient was admitted to ICU and later discharged without returning to another ICU). Any patients with multiple ICU stays were excluded from the study. Patient records were between 2001 and 2012 at the Beth Israel Deaconess Medical Centre in the United States (U.S.), which is a world-class teaching hospital of Harvard Medical School with 77 ICU beds located in central Boston [19, 20].

We included patients with any diagnosis from: transfers to ICU from external hospital wards, transfers from wards within the hospital and direct admissions from the emergency department. We did not exclude patients with Do Not Attempt Resuscitation (DNAR) orders.

**Fig. 1:**
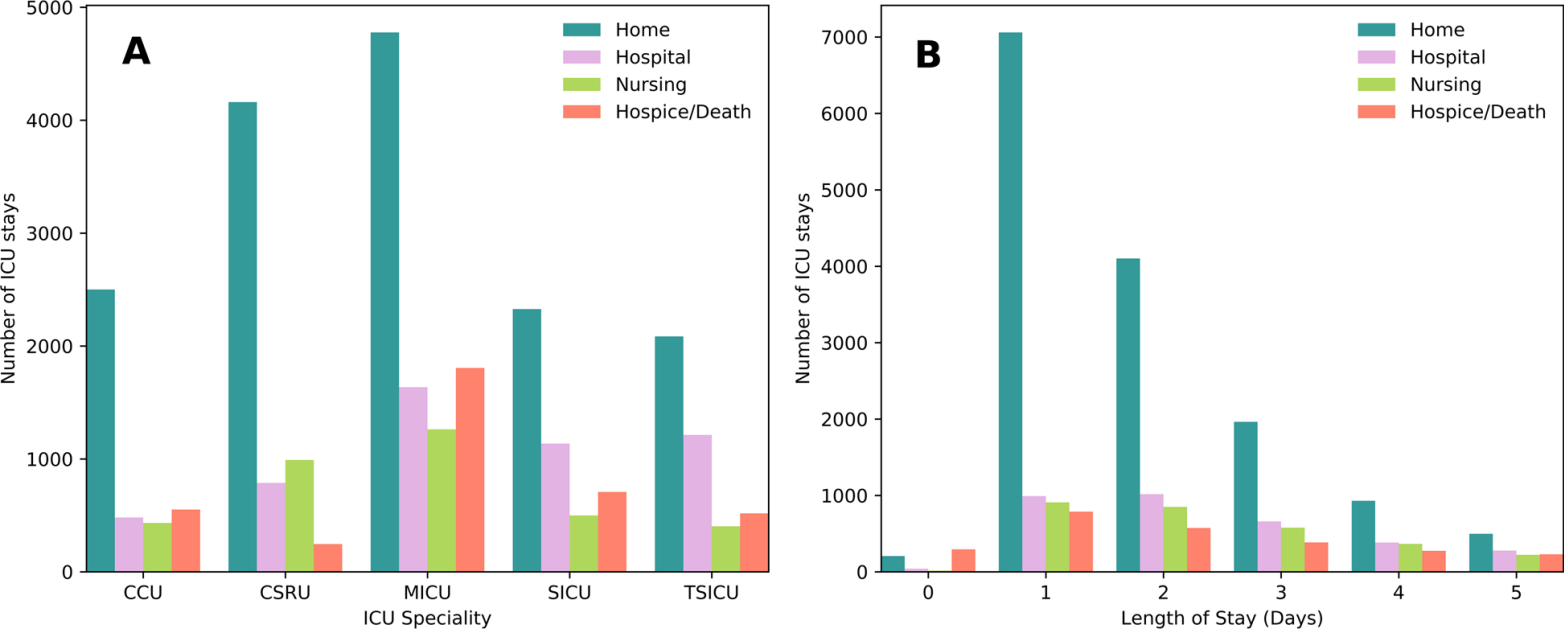
Intensive Care Unit (ICU) summary for Cardiac Care Unit, Cardiac Surgery Recovery Unit, Medical ICU, Surgical ICU, Trauma Surgical ICU. **a** ICU specialty and discharge. **b** Duration of critical care (up to 5 days shown)

While MICU had the largest number of discharges to home (Fig. 1a), this only represents 50.39% of the MICU admissions, whereas proportionally more patients were discharged to home from CSRU (67.26%) and CCU (63.02%). This is likely to be because the most acutely ill patients were admitted to the MICU.

TSICU (28.75%) and SICU (24.30%) had the largest proportions of their respective patients who were discharged to hospital wards, CSRU (16.02%) had the largest proportion discharged to a nursing facility and the most deaths occured proportionally within the MICU (19.05%).

Patients with 16 ≤*age*≤89 were included in the analysis who had a single admission to an ICU, resulting in a cohort of 28,523 patients (16,517 male, 12,006 female). The overall mean (95% CI) length of stay (LOS) was 3.89 days (3.83–4.0) and the LOS range was 104.25 days (Fig. 1b). The mean LOS for patients discharged home was the lowest (2.4) whereas it was longest for those patients discharged to hospital (7.0). Patients discharged to a nursing facility had a shorter mean stay (3.8) compared to those who died (5.9).

### Outcome

The main outcome of interest for this study was the discharge location for a patient leaving the ICU. From the patient records, there were 17 unique discharge location types for patients, which we grouped according to the similarity of patient care level which we infer from the discharge location. These unique discharge locations were collated and are defined in Table 1. The location to which the ICU patient was discharged to is a surrogate for the patient’s physical status after discharge. The categories provide the only measure available for early outcome from the ICU database. It is important to note that patients grouped by discharge location had varied levels of recoveries, but that the patients within their respective discharge location group shared more similarities with other members of that discharge location group in terms of clinical support than with patients from another discharge location group. To complement this, Table 2 includes a comparison of the discharge locations with a breakdown of the ICU Length of Stay (LOS) which shows distinct similarities across the discharge locations, as well as unique differences across the ICU specialties. In Table 2 we note that CCU and CSRU patients had the lowest IQR for LOS compared to the other ICU specialties.

**Table 1:** Discharge Locations for Intensive Care Unit (ICU) patients and the implied hospital care needs

Discharge Location (care level)	Details
Home (low)	Patients who were discharged home with no support, or with support, and those who left against advice. This includes those directly discharged from the ICU to home, as well as those who were transferred out of ICU to a hospital ward for a short recovery period, before a final discharge to home is recorded. Recovery period mean (±*SD*) is 6 (4.6) days.
Hospital Ward (medium)	Patients who were transferred to other wards in the hospital, or to another external hospital for an extended period. These patients remain in the hospital environment on a permanent or semi-permanent basis.
Nursing/ Primary Care (high)	Patients who were discharged to a skilled nursing facility.
Hospice/ Death (palliative)	Patients who were discharged to a medical hospice, home hospice, or death.

**Table 2:** Patient discharge location Length of Stay (LOS) comparison for different Intensive Care Units (ICUs): Cardiac Care Unit, Cardiac Surgery Recovery Unit, Medical ICU, Surgical ICU, Trauma Surgical ICU

		Median (*IQR*) Length of Stay (LOS) in days for each Discharge Location				
Cohort	Patients *n*	Home	Hospital Ward	Nursing/ Primary Care	Hospice/Death	Total
CCU	3,967	1.75 (1.74)	4.10 (6.89)	2.98 (3.30)	3.57 (6.33)	2.05 (2.76)
CSRU	6,185	1.47 (1.52)	3.98 (7.64)	2.49 (2.84)	4.07 (8.85)	2.05 (2.11)
MICU	9,482	1.69 (1.73)	2.88 (5.76)	2.36 (2.63)	3.22 (5.84)	2.00 (2.82)
SICU	4,670	1.77 (1.84)	3.17 (7.25)	2.78 (4.06)	2.81 (5.70)	2.11 (3.38)
TSICU	4,219	1.59 (1.69)	3.54 (8.09)	2.00 (2.48)	2.75 (5.50)	2.00 (3.27)
All	28,523	1.67 (1.70)	3.31 (7.10)	2.55 (2.88)	3.13 (5.94)	2.04 (2.80)

Patients discharged to a hospice account for only *n*=305 (1.06%) of the cohort, and this small proportion of patients cannot be effectively predicted against the other larger discharge locations. Therefore we decided to combine the hospice patients with the death location instead of with Nursing/Primary Care location by looking at the patient records and identifying a ‘Date of Death’ that was recorded outside of their hospital admission, provided by the social security database, which occurs within 1 year of their ICU discharge date. Therefore, in the medium-long term outlook, these patients belonged in the death discharge category.

### Variables

**Fig. 2:**
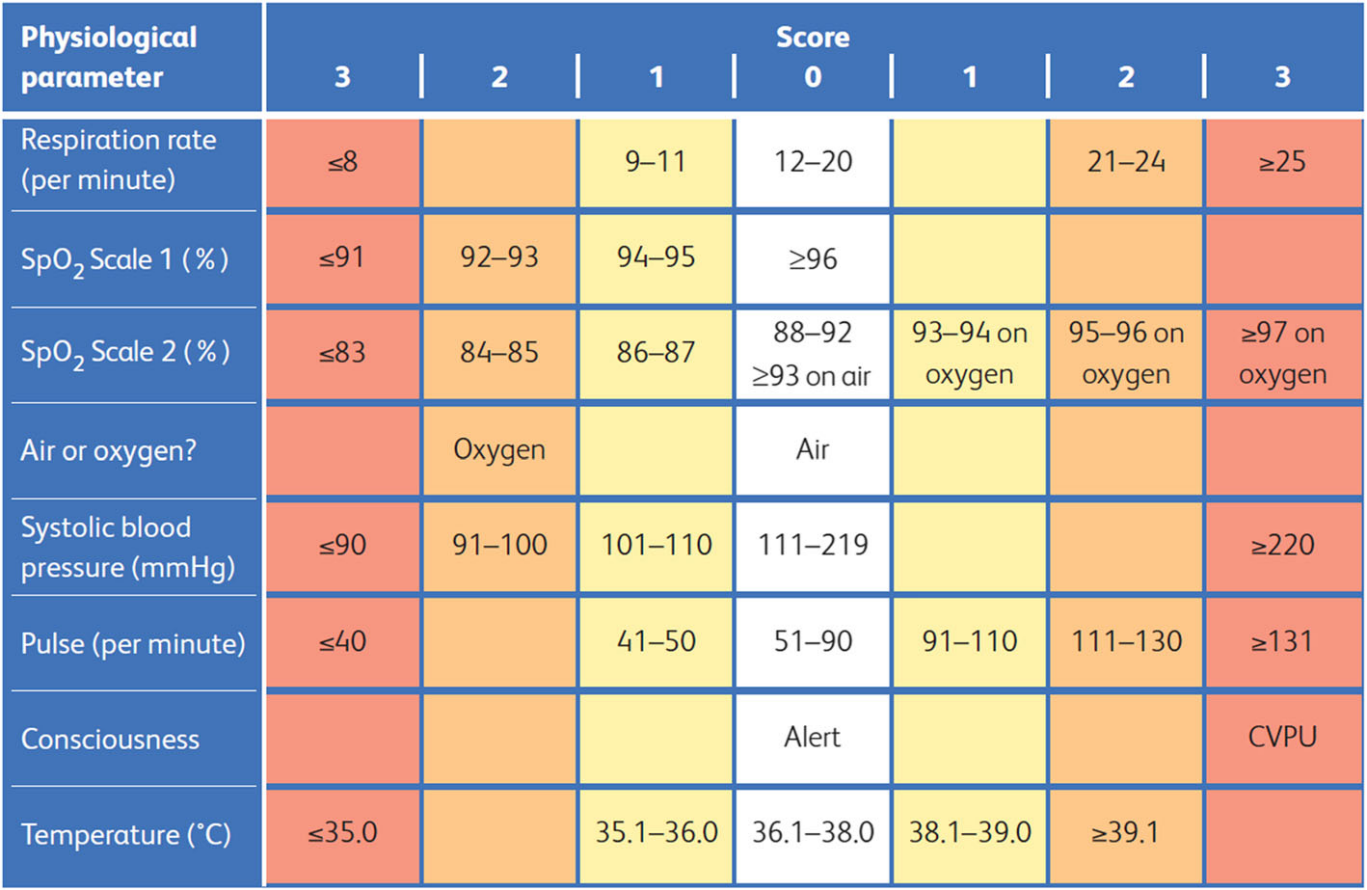
The National Early Warning Score (NEWS)(weightings for the seven parameters) Figure reproduced from the Royal College of Physicians [1]

The individual NEWS parameters and aggregate NEWS were derived hourly (Fig. 2) for the first 24h following admission. Where multiple recordings were taken within the same hour, the NEWS calls for the most severe derangement of a vital sign to be the one recorded [1]. Where a measurement was not taken for any particular hour, we carried the previous hour measurement forward.

2,723,055 measurements were recorded for the first 24h corresponding to the vital signs that comprise the NEWS. Where a patient’s conscious level was assessed with Glasgow Coma Scale (GCS) instead of Alert, Confusion, Verbal, Painful, Unresponsive (ACVPU), the GCS value was converted to an ACVPU equivalent: GCS 15 = A, GCS 14 or below = CVPU.

Collinearity between variables was included for improving accuracy for discharge prediction (i.e. using the minimum, median and maximum NEW score for a patient’s respiration rate within the 24h period). Thus, 7 component NEWS parameters and aggregate NEWS provided 24 parameters for the NEWS across the first 24h of admission. We did not modify the NEWS weightings or triggers to maximise its ability to discriminate any particular outcome.

### Missing Glasgow Coma Scale (GCS)

Most variables were available during the initial 24h of admission, except for GCS values (44% missing). The collection of GCS measurements were clinically very likely to be done with less frequency than other vital signs such as heart rate or blood pressure. For example, comparing hourly vital sign measurements will lead to missing GCS scores as GCS may be measured every other hour, or on an ad hoc basis, while other vital signs are measured continuously. This is a characteristic presented in other patient datasets as well [21]. The high proportion of missing values may also be due to the variability in the use of GCS recording in some ICU populations, or if there was no interface to import the nursing flow-sheet data from the primary electronic medical record, which will result in missing GCS values.

For our analysis, where no GCS value was available to calculate the consciousness score (Fig. 2), we tested several standard techniques for data imputation and found the best performing three to be: logistic regression, decision tables and a multilayered perceptron. We used the logistic regression method for imputation.

For imputation we used: Heart Rate, Temperature, Respiration Rate, O2 Saturation, O2 Delivery and Blood Pressure (Systolic). These were at an hourly resolution (where multiple recordings were taken within the same hour, we take the most severely deranged value to be the one recorded). We also include the minimum, median and maximum for each vital sign NEW score across the first 24 h. We did not include other patient specific variables such as age, gender or ICU type.

A total of 383,349 complete ACVPU (GCS) measurements were available for training (in a 70/30 hold-out) and the evaluation of imputation model was by AUROC. Once completed, the model was applied to the missing ACVPU (GCS) with 301,202 (44%) imputations. We compared distributions and summary statistics of the ACVPU for the newly imputed set against the complete set used for training and evaluation, to ensure that the imputations were representative and consistent.

As it is more likely that disturbances in multiple physiological parameters occur in unison rather than in isolation, we were able to predict the ACVPU value for patients at the 24 h time period with an Area Under the Receiver Operating Characteristic (AUROC) curve (95% CI) of 0.985 (0.978–0.992). Thus, we ensure that the consciousness score for every patient was a faithful representation of their physiological condition, instead of simply replacing missing values by other averaging methods. We sampled by a shuffled random 10 fold cross-validation, and overcame the imbalanced discharge classes by class weighting and Synthetic Minority Oversampling Technique (SMOTE) [22]. Data manipulation and analysis were performed with Python version 3.6 [23].

### Evaluation

Evaluation of performance was by the consideration of the AUROC curve. An AUROC value is measured between 0 and 1, where a value of 0.5 indicates that the discrimination of discharge location for the NEWS was no better than random chance. Thus, we consider a reasonable discrimination as being indicated by an AUROC value of 0.700–0.800 and good discrimination by values above 0.800. We compared NEWS performance under each ICU population by the paired AUROC method [24] to test significance (*p*<0.05) of differences between AUROC.

## Results

The mean (±*SD*) vital signs for the first 24 h of all patient admissions were: respiration rate 18.7 (5.4)breaths min^-1^; SpO2 96.4 (2.3)%; systolic BP 119.2 (20.6)mmHg; heart rate 85.3 (17.8)beats min^-1^; temperature 37.0 (0.9) ^∘^C. The number of observations at different levels of consciousness were: Alert (11,539), CVPU (16,985).

**Fig. 3:**
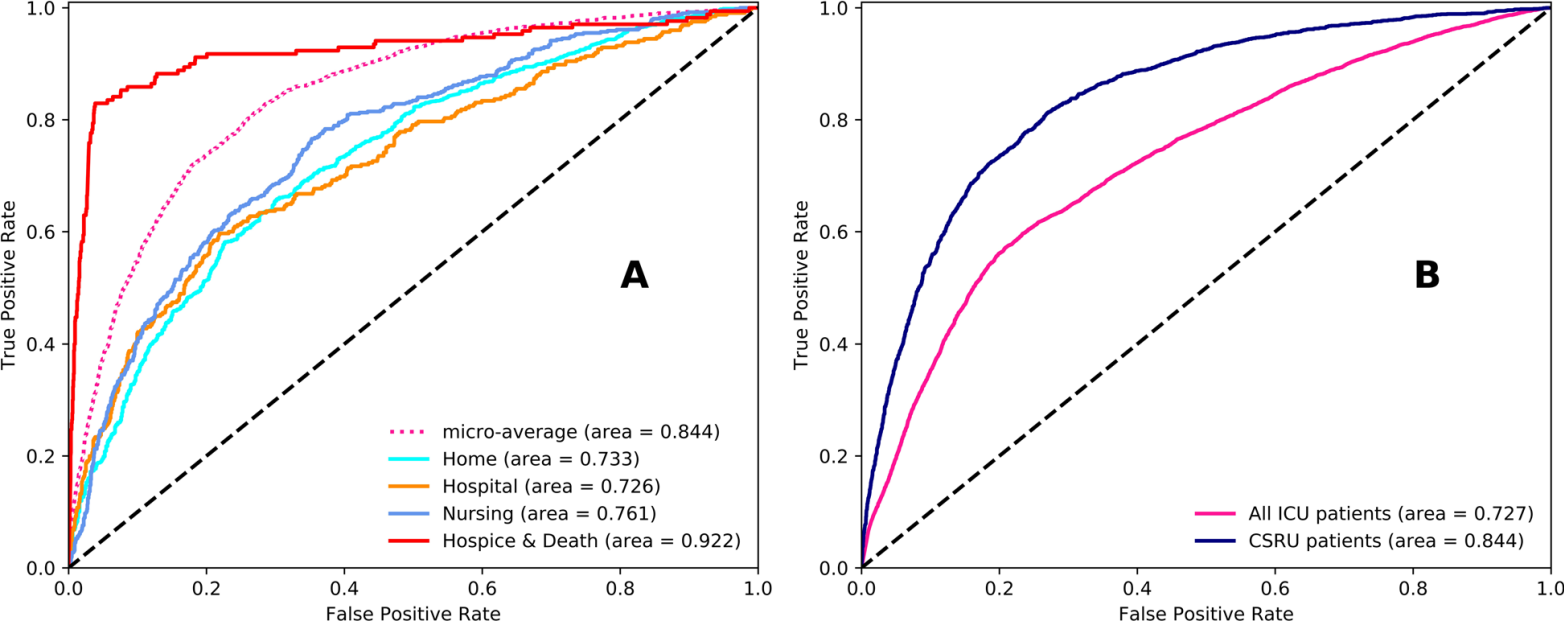
AUROC curve for The National Early Warning Score (NEWS) within Cardiac Surgery Recovery Unit (CSRU) and general Intensive Care Unit (ICU) population. **a** NEWS discrimination within CSRU. **b** NEWS discrimination for CSRU and total ICU population

The AUROC values, measuring the discrimination of the NEWS for discharge location, show large differences between the ICU specialties and overall ICU population (Table 3), with best performance in CSRU (Fig. 3b). NEWS discrimination was good for CSRU and CCU patients and reasonable for MICU, SICU and TSICU. Discrimination was best when the NEWS was used within each different ICU compared to in general use, which may be due to the physiological similarities within patients in each ICU and physiological differences of patients between ICU specialties.

**Table 3:** National Early Warning Score (NEWS) discrimination across different Intensive Care Units (ICUs): Cardiac Care Unit, Cardiac Surgery Recovery Unit, Medical ICU, Surgical ICU, Trauma Surgical ICU

Cohort	AUROC	95% CI	*p*-Value
CCU (*n*=3967)	0.829	0.821–0.837*	<0.001
CSRU (*n*=6185)	0.844	0.838–0.850*	<0.001
MICU (*n*=9482)	0.778	0.767–0.791	<0.001
SICU (*n*=4670)	0.775	0.762–0.788	<0.001
TSICU (*n*=4219)	0.765	0.751–0.773	<0.001
All patients (*n*=28523)	0.727	0.709–0.745	<0.004

^*^AUROC that are significantly higher (95% confidence intervals do not overlap)

The differences between AUROC for NEWS were significant (95% CI do not overlap) in CCU and CSRU patients. Within CSRU, NEWS discrimination was reasonable across Home, Hospital and Nursing with good discrimination for Death (Fig. 3a, Table 4).

**Table 4:** National Early Warning Score (NEWS) discrimination for each location within the Cardiac Surgery Recovery Unit (CSRU)

CSRU	AUROC	95% CI	*p*-Value
Home (*n*=4,160)	0.733	0.720-0.746	<0.003
Hospital (*n*=788)	0.726	0.707-0.745	<0.001
Nursing (*n*=991)	0.761	0.750-0.772*	0.034
Hospice/Death (*n*=246)	0.922	0.911-0.933*	<0.001

^*^AUROC that are significantly higher (95% confidence intervals do not overlap)

## Discussion

The NEWS had good discrimination within CCU and CSRU patients with reasonable discrimination in MICU, SICU, TSICU and in general for all ICU patients.

A potential explanation for the significant difference in discrimination of the NEWS for CSRU patients is that these patients may not have been as systemically sick as other patients in the other ICU specialties: whilst CSRU patients do have severely disordered cardiac function, they underwent corrective surgery, and would not have been scheduled for surgery if they did not have otherwise good physiological status. Therefore it could be inferred that this patient group were probably healthier overall. This corroborates with the earlier commentary (Fig. 1a) that CSRU had proportionately the lowest number of patients who died or went to a hospice and the highest number who were discharged home compared to the other ICU specialties. Additionally, Table 2 shows that both CCU and CSRU had the lowest IQR for Length of Stay (LOS) compared to the other ICU specialties, indicating that these were a more consistent cohort in comparison to the other ICUs.

We found a statistical significance (*p*<0.05) in difference of mean respiration, blood pressure, heart rate and GCS in ICU specialties, when comparing CSRU and CCU with one another as well as with the other ICUs (where MICU, SICU and TSICU were grouped together) as presented in Table 5. We also found statistical significance (*p*<0.05) in the difference between the mean O2 saturation for the other ICU specialties against CSRU and CCU in Table 5. This therefore helps with the interpretation of how the NEWS is able to discriminate better in these cohorts, as there was a difference in the underlying vital signs which translates to different levels of NEWS.

**Table 5:** Intensive Care Unit (ICU) vital sign means for Cardiac Surgery Recovery Unit, Cardiac Care Unit and other ICUs

Vital Sign	CSRU mean (95% CI)	CCU mean (95% CI)	Other ICUs mean (95% CI)
Respiration (per min)	18.82 (18.65–18.99)*	20.25 (20.13–20.36)*	20.79 (20.74–20.85)*
O2 Saturation (%)	96.13 (96.04–96.23)	96.09 (96.02–96.16)	95.87 (95.85–95.90)*
Blood Pressure (mmHg)	111.93 (111.36–112.50)*	109.10 (108.67–109.54)*	117.24 (117.05–117.43)*
Heart Rate (bpm)	86.82 (86.28–87.37)*	87.86 (87.46–88.26)*	91.38 (91.21–91.54)*
GCS	7.86 (7.60-8.12)*	8.98 (8.80-9.16)*	8.46 (8.39-8.52)*
Temperature ^∘^C	36.84 (36.79-36.88)	36.81 (36.76–36.86)	36.89 (36.87–36.91)

^*^95% confidence intervals do not overlap

One might expect that a patient who was close to death would have significantly different physiological signals compared to those who are alive or go on to recover, and we see that comparing patients with a Hospice/Death category with the other discharge locations collectively for the vital signs, that there was a statistically significant (*p*<0.05) difference in vital sign means, shown in Table 6. This helps to illustrate how the NEWS, which is a method of collating these vital signs, could distinguish between those in the hospice/death category with higher discrimination than other categories.

**Table 6:** Vital sign means for Hospice/Death cohort

Vital Sign	Hospice/Death mean (95% CI)	Other Discharge Location mean (95% CI)
Respiration (per min)	20.60 (20.55–20.64)*	18.42 (18.41–18.42)*
O2 Saturation (%)	95.92 (95.90–95.95)*	96.48 (96.47–96.48)*
Blood Pressure (mmHg)	115.78 (115.61–115.94)*	118.28 (118.25–118.31)*
Heart Rate (bpm)	90.61 (90.46–90.76)*	83.91 (83.88–83.94)*
GCS	8.48 (8.42–8.54)*	12.44 (12.42–12.45)*
Temperature ^∘^C	36.87 (36.85–36.89)*	37.06 (37.05–37.06)*

^*^95% confidence intervals do not overlap

The loss of discrimination ability of the NEWS when tested over the whole ICU population could be due to the significant physiological differences between each ICU population, which is lost in the NEWS weightings. In keeping with the National Institute for Health and Care Excellence (NICE) guidelines [25], the NEWS could be optimised for the discharge decision-making process by adjusting the weightings and not by additional variables. This may allow for improved discrimination ability prior to use as an overall tool in the discharge decision-making process, irrespective of ICU specialty and even beyond the ICU domain.

The performance of the NEWS for discrimination of death was similar to other work on the NEWS [26] and other models for discriminating for the outcome of death [16]. Other discharge models [16] used over 20 patient measurements, whereas the NEWS only uses 7 parameters. Additionally these other discharge models were developed with the intention of being calculated through computer systems and not by clinicians at the bedside. Therefore these models do not align with the motivations [1, 25] of unifying scoring systems which led to the NEWS. Other discharge models also include a risk factor for readmission to ICU [16], and as this is a study on a single-stay ICU patient population, we were unable to include such a risk factor in our analysis.

This study has several strengths: this is the first to compare the NEWS against multiple discharge locations and for its application in discharge planning. We used over 2.7 million vital sign observations across 28,523 patients from different ICU specialties to present a new and operationally useful outcome for any hospital, i.e. who will be discharged to what location within 24h of any admission. This allows opportunities for appropriate management of resources, and overall improvement in the care pathway of each patient.

There are several limitations to note: The patient population is obtained from a single hospital in the U.S. and the results may not be generalisable to other settings. The aim of this research was to focus on the capability of the NEWS in addressing an area that is amenable to change, specifically the ability of the hospital’s systems to adapt in order to plan clinical resource allocation more effectively.

While we exclude multiple-stay ICU patients, we cannot be certain that a patient did not attend an ICU outside of the study hospital, or even before the study period began. Therefore this is a potential factor which could have had an effect on the results of this study.

Approximately 1% (*n*=30,220) of patient vital sign measurements were recorded at a chart time that was before that patient’s admission time into the ICU. This was most likely due to administration error, where the patient was physically in the ward and connected to the bedside monitors, but not administered onto the system yet. In these instances we shifted the individual patient admission time back to when the first measurement was taken for this subset to utilise these measurements accordingly.

With the aim of risk assessment in acutely ill patients, regardless of their location at any point in time, it is imperative to capture patient relevant data. Thus, using the earliest measurements obtainable is the most valid approach to answer the research question. We also believe no tool should be restricted to the ICU admission, and it is our view that we would ultimately like to see every hospital admission dynamically graded for predicted outcomes from the point of admission to the ER.

Due to the retrospective design, all discharges from the ICU were made at the discretion of the attending physician. No information is available to confirm which, if any, discharge planning tools were used for the patients. Additionally, there are other competing factors such as location, disease or family support which would affect discharge location, but in this study were not explored. The underlying motivation of clinicians to recommend, or have recommended, a specific discharge location is another consideration which we were unable to address in this work.

Guidelines [1] for the implementation of the NEWS encourage that in the absence of a measurement, to assign the most severe score for that NEWS parameter. For example, if the temperature is not measured for a patient, assign this a NEW score of 3. Therefore, it would be entirely reasonable when dealing with the missing ACVPU scores in this study (derived from GCS) to assign these a NEW score of 3.

Our purpose of imputing the missing ACVPU scores was motivated by the desire to ensure that any testing period was as faithful a representation as possible of a patient’s true physiological condition at that period in their admission. We suggested that disturbances in physiological parameters are related, so if one or more physiological parameter was significantly deranged, then it is more likely that ACVPU would also be deranged, *mutatis mutandis* when all physiological parameters are NEW score 0, ACVPU would likely be NEW score 0.

Thus, there are different methods of imputation for missing NEWS parameters, which could lead to different outcomes in NEWS performance.

We do not currently provide new clinical triggers or weightings for the NEWS. In the context of recommending a discharge we suggest that further comparative studies are required with the view that a complementary set of weightings and triggers can be developed for the NEWS which will allow for it’s universal integration and optimisation in the discharge decision-making process, while keeping within the NICE guidelines [25].

Standardisation of the discharge process in ICUs is a highly desirable objective. Hospitals should not have different discharge systems or methods for their patients, while treating similar patient populations and diseases.

## Conclusions

We have demonstrated that the National Early Warning Score (NEWS) was able to discriminate a patient by discharge location within 24h of admission to any ICU specialty, indicating that care providers could potentially have an idea of future discharge needs very early into a patient’s admission, thus reducing the likelihood of both premature discharge and discharge delay by allowing care providers adequate time to plan accordingly.

It is an important planning requirement that future patient needs are predicted and care demands met. By accurately predicting patient discharges, patient needs can be defined earlier and provisions can be made in anticipation for their timely discharge, thus allowing the services beyond ICU to be connected and streamlined.

## Data Availability

The data that support the findings of this study are available from Pollard et al. & Johnson et al. [19, 20] but restrictions apply to the availability of these data, which were used under license for the current study, and so are not publicly available. Data are however available from the authors upon reasonable request and with permission of Pollard et al. & Johnson et al. [19, 20].

## Abbreviations

ACVPU: Alert, confusion, verbal, painful, unresponsive
AUROC: Area under the receiver operating characteristic
CCU: Coronary care unit
CI: Confidence interval
CSRU: Cardiac surgery recovery unit
DNAR: Do not attempt resuscitation
GCS: Glasgow coma scale
ICU: Intensive care unit
IQR: Interquartile range
LOS: Length of stay
MICU: Medical intensive care unit
NEWS: National early warning score
NHS: National health service
NICE: National institute for health and care excellence
SICU: Surgical intensive care unit
SMOTE: Synthetic minority oversampling technique
TSICU: Trauma/ surgical intensive care unit
U.K.: United Kingdom
U.S.: United States
